# Effectiveness of robust optimization against geometric uncertainties in TomoHelical planning for prostate cancer

**DOI:** 10.1002/acm2.13881

**Published:** 2022-12-28

**Authors:** Takayuki Yagihashi, Kazumasa Inoue, Hironori Nagata, Masashi Yamanaka, Akihiro Yamano, Shunsuke Suzuki, Wataru Yamakabe, Naoki Sato, Motoko Omura, Tatsuya Inoue

**Affiliations:** ^1^ Department of Medical Physics Shonan Kamakura General Hospital Kamakura City Kanagawa Japan; ^2^ Graduate School of Human Health Sciences Tokyo Metropolitan University Arakawa‐ku Tokyo Japan; ^3^ Medical Physics Laboratory, Division of Health Science, Graduate School of Medicine Osaka University Suita‐shi Osaka Japan; ^4^ Graduate School of Engineering Kyoto University Nishikyo‐ku Kyoto Japan; ^5^ Department of Radiation Oncology Shonan Kamakura General Hospital Kamakura City Kanagawa Japan; ^6^ Department of Radiation Oncology Juntendo University Bunkyo‐ku Tokyo Japan

**Keywords:** helical tomotherapy, intensity‐modulated radiotherapy, localized prostate cancer, minimax robust optimization

## Abstract

**Background:**

Geometrical uncertainties in patients can severely affect the quality of radiotherapy.

**Purpose:**

We evaluated the dosimetric efficacy of robust optimization for helical intensity‐modulated radiotherapy (IMRT) planning in the presence of patient setup uncertainty and anatomical changes.

**Methods:**

Two helical IMRT plans for 10 patients with localized prostate cancer were created using either minimax robust optimization (robust plan) or a conventional planning target volume (PTV) margin approach (PTV plan). Plan robustness was evaluated by creating perturbed dose plans with setup uncertainty from isocenter shifts and anatomical changes due to organ variation. The magnitudes of the geometrical uncertainties were based on the patient setup uncertainty considered during robust optimization, which was identical to the PTV margin. The homogeneity index, and target coverage (TC, defined as the V100% of the clinical target volume), and organs at risk (OAR; rectum and bladder) doses were analyzed for all nominal and perturbed plans. A statistical *t*‐test was performed to evaluate the differences between the robust and PTV plans.

**Results:**

Comparison of the nominal plans showed that the robust plans had lower OAR doses and a worse homogeneity index and TC than the PTV plans. The evaluations of robustness that considered setup errors more than the PTV margin demonstrated that the worst‐case perturbed scenarios for robust plans had significantly higher TC while maintaining lower OAR doses. However, when anatomical changes were considered, improvement in TC from robust optimization was not observed in the worst‐case perturbed plans.

**Conclusions:**

For helical IMRT planning in localized prostate cancer, robust optimization provides benefits over PTV margin–based planning, including better OAR sparing, and increased robustness against systematic patient‐setup errors.

## 1 INTRODUCTION

Radiotherapy is a standard treatment option for patients with localized prostate cancer, conferring a long‐term tumor‐control probability equivalent to that of prostatectomy.^1^ ^,^ ^2^ Intensity‐modulated techniques are frequently used to produce better dose conformity to the tumor and decrease the dose to the organs at risks (OARs), such as the rectum and bladder, in radiotherapy. However, due to the high dose conformity and sharp dose gradient, intensity‐modulated radiotherapy (IMRT) is more sensitive to geometric uncertainties in patients, including setup errors, tumor motion, and anatomical changes. These uncertainties can potentially cause greater OAR doses and underdoses of the target volume. A conventional approach to compensate for the effect of uncertainties in IMRT is to apply a planning target volume (PTV) margin to the clinical target volume (CTV).^3^ The use of a PTV margin is based on the assumption that the dose distribution is invariant; however, setup errors and anatomical changes can affect the shape of the dose distribution.

A robust optimization method has been developed to handle geometric uncertainties in treatment planning without the use of conventional margins.^4^ ^,^ ^5^ In the robust optimization process, shifting beam coordinates in computed tomography (CT) are performed to concurrently calculate the dose distributions for multiple realistic scenarios that consider geometric uncertainties. Subsequently, the objective function of worst‐case dose distribution from each scenario dose is optimized in each iteration, resulting in a plan that is robust to geometric uncertainties. Several studies have demonstrated that intensity‐modulated proton therapy (IMPT) and volumetric modulated arc therapy (VMAT) plans with robust optimization can outperform PTV‐based plans with regard to guaranteeing robust target coverage and sparing normal tissue in the lung,^6^ ^–^ ^8^ head and neck,^9^ ^–^ ^11^ and breast cancers^12^ ^,^ ^13^ that are especially sensitive to organ motion and anatomical changes.

Tomotherapy (Accuray; Sunnyvale, CA, USA) is a helical intensity‐modulated radiation therapy machine that delivers radiation beams with 360° gantry rotation while translating the treatment couch.^14^ ^,^ ^15^ Unlike standard linear accelerators, this machine enables changes in the angle and intensity of the beam according to cranial‐caudal coordinates, resulting in dose distribution and delivery with excellent target coverage and OAR sparing. The dosimetric efficacy of tomotherapy compared to other IMRT techniques, such as VMAT, has been reported for prostate cancer^16^ ^–^ ^19^ and other treatment sites.^20^ ^–^ ^26^ Recently, the RayStation treatment planning system (TPS) (RaySearch Lab; Stockholm, Sweden) implemented a new module that performs dose calculations for tomotherapy. The TPS is also available for robust optimization in treatment planning for worst‐case scenarios. However, there are only a few investigations into the clinical benefits of robust optimization for tomotherapy.^13^ ^,^ ^27^ This study is the first to compare robustly optimized plans with PTV‐based plans, in terms of target coverage and OAR‐sparing efficacy considering patient setup errors and anatomical changes, in patients with localized prostate cancer treated with helical IMRT.

## 2 MATERIALS AND METHODS

### 2.1 Patient selection and structure contouring

This retrospective planning study was approved by the institutional review board of Shonan Kamakura General Hospital (No. 1955). We randomly selected 10 patients with localized low‐ or intermediate‐risk prostate cancer who received definitive radiotherapy with a TomoTherapy Radixact unit (Accuray; Sunnyvale, CA, USA), using helical IMRT consisting of a prescription of 60 Gy to the target volume in 20 fractions. The clinical tumor stage was T1c‐3a. No patient had evidence of enlarged lymph nodes on CT/magnetic resonance imaging, and bone scans revealed no evidence of distant bony metastases. All patients had previously undergone CT scanning on a Siemens 20‐slice CT scanner (Somatom Confidence; Siemens, Germany) with a reconstruction resolution of 0.967 × 0.967 × 2 mm^3^ in the axial helical mode. The target volume and OARs (rectum and bladder) were delineated using the CHHiP protocol, version 8,^28^ on the CT images in RayStation version 10A. The PTV consisted of the CTV with 8 mm margins in each direction, except 5 mm was used posteriorly. In addition, two ring structures around the PTV were created to control the steep dose‐falloff for the volume outside the PTV during the optimization process. The first ring ranged to 0.3 cm outside the PTV; the second ring started at the first ring and ranged 0.8 cm outside the PTV. The characteristics of the patients enrolled in the study are summarized in Table 1.

**TABLE 1:** Patient characteristics

Patient	Age	cT stage	CTV (cc)	PTV (cc)	Bladder (cc)	Rectum (cc)
1	79	T1c	55.7	135.8	114.0	40.1
2	71	T3a	23.8	80.6	148.1	46.7
3	57	T3a	21.3	70.6	176.5	81.4
4	76	T1c	25.5	77.3	255.0	33.9
5	75	T1c	24.2	78.2	171.7	42.2
6	76	T2a	24.3	75.4	185.9	61.3
7	87	T2c	26.3	77.4	230.7	52.8
8	85	T1c	23.5	74.7	220.1	45.9
9	87	T2b	59.9	142.3	207.2	38.2
10	73	T2c	21.1	71.1	161.2	84.8

### 2.2 Treatment planning

Based on the patient CT and structure dataset used for clinical treatments, two types of nominal helical IMRT plans were created in RayStation: a PTV‐based conventional optimization (PTV plan) and a robust optimization (robust plan).

#### 2.2.1 PTV plan

The planned isocenter was first placed at the centroid of the CTV. The PTV plans were then optimized to achieve a prescription dose of 60 Gy in 20 fractions that covered ≥95% of the PTV but <107% of the prescription dose with 6 MV. The dose constraints for the bladder and rectum were based on previously published recommendations^29^ ^,^ ^30^: rectum V30Gy < 50%, rectum V57Gy < 15%, bladder V40Gy < 50%, bladder V50Gy < 30%, and bladder V60Gy < 5%. The objective weights were varied to decrease the OAR dose as much as possible, without compromising the PTV criteria. Dose‐falloff parameters of 60 to 54 Gy and 54 to 30 Gy were set for the first and second rings, respectively. Each plan was optimized using 60 iterations. The calculation parameters were a 2.5‐cm field width, 0.287 pitch, and 1.8 delivery time factor. The delivery time factor *x* is a new parameter implemented in RayStation for tomotherapy; the delivery time cannot exceed *x* multiplied by *t*, where *t* is the delivery time when the leaf‐open times are uniform and scaled such that the average target dose equals an estimate of the prescription.^31^ The final dose was calculated using a collapsed cone convolution algorithm with a grid size of 2 mm.

#### 2.2.2 Robust plan

The robust plans were optimized to achieve a prescription dose of 60 Gy in 20 fractions that covered at least 95% of the CTV but were < 64.2 Gy in the minimax optimization function.^4^ To mimic the PTV margin, the patient‐setup uncertainties were simulated by shifting the plan isocenter by 8 mm in the left–right (LR), superior–inferior (SI), and anterior directions, and 5 mm in the posterior direction during robust optimization. The objective function of the worst‐case scenario from all the scenario doses, including the nominal (no‐shift), was minimized. For comparison purposes, the beam geometry and the planning and optimization parameters were kept identical for the PTV and robust plans, except for the target volume. The dose constraints and objective for the two ring structures were identical, although the target volume in the robust plans was smaller than that in the PTV plans.

### 2.3 Evaluation of robustness

After creating each nominal plan, the robustness of the plans against the two types of uncertainty was evaluated.

#### 2.3.1 Setup error

**FIGURE 1:**
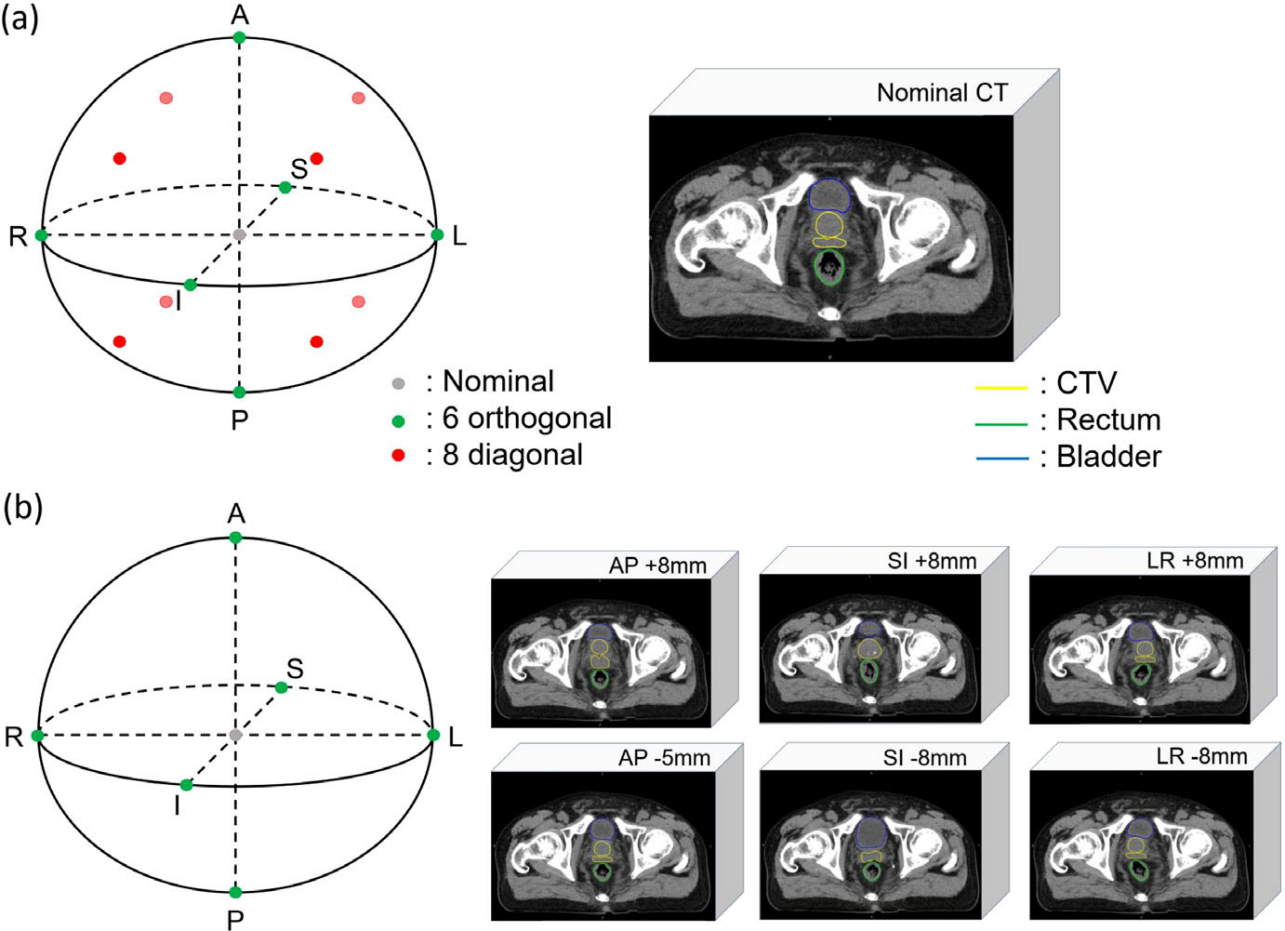
Schematic diagram for robustness evaluation against (a) setup error, and (b) anatomical change

The robustness against setup error was assessed by shifting the plan isocenter in 14 directions (Figure 1a): 8 mm in the LR, SI, and anterior directions, 5 mm in the posterior direction, and their combinations (8/5 mm setup error). These correspond to the same scenarios in the robust optimization process. To rank the robust plans, smaller and greater shifts were assessed: 5 and 10 mm in the LR, SI, and anterior directions, 3 and 7 mm in the posterior direction, and their combinations (5/3 mm, and 10/7 mm setup errors, respectively). Considering these setup errors, the dose distributions were recalculated on the same CT image. In total, 56 perturbed dose distributions were created for each patient (28 for the robust plan and 28 for the PTV plan).

#### 2.3.2 Anatomical change

To assess the robustness of each nominal plan to anatomical changes, the CT images were deformed by the CTV in six directions (Figure 1b): 8 mm in the LR, SI, and anterior directions, and 5 mm in the posterior direction. These were created from the original planning CT by simulating organ motion functionality based on a non‐rigid deformable image registration algorithm in RayStation.^32^ The dose distributions were recalculated for the six CT images using the same planning conditions for each nominal plan. Consequently, 12 perturbed dose distributions were created for each patient (six for the robust plan and six for the PTV plan).

### 2.4 Evaluation metrics

The target coverage (TC) and the homogeneity index (HI)^33^ were calculated for all nominal and perturbed dose distributions in both the robust and PTV plans.

TC was defined by a CTV receiving the prescription dose of 60 Gy (V100%). The HI was calculated using Equation 1:

(1) $$\mathrm{HI}=\frac{D_{2\%}-D_{98\%}}{D_{50\%}}$$

where D2%, D98%, and D50% are the doses covering 2%, 98%, and 50% of the CTV, respectively. The rectum V30Gy and V57Gy, bladder V40Gy, V50Gy, and V60Gy, and expected irradiation times were also evaluated. The paired *t*‐test was used to calculate two‐tailed *p*‐values using Matlab 2020b (Mathworks Inc., Natick, MA, USA). Differences in the evaluation metrics between the robust and PTV plans were considered statistically significant if *p* < 0.05.

## 3 RESULTS

The D2%, D98%, HI, and TC of the CTV from the robust and PTV nominal plans are summarized in Table 2. All plans had clinically adequate CTV coverage (D98% > 57 Gy [95%] and D2% < 64.2 Gy [107%]). The robust plan showed worse HI and TC, compared with the PTV plan. The robust plans spared the rectum V57Gy, and bladder V40Gy, V50Gy, and V60Gy, significantly better in all patients. However, it was difficult to achieve the rectum V57Gy dose constraint for the robust plans of one patient and PTV plans of two patients because of the small volume. Likewise, the bladder V60Gy dose constraint was not met for the robust plans of three patients and the PTV plans of seven patients. The expected delivery time data are listed in Table 2. Compared with the PTV plans, the robust plans reduced the time by 3.0–21.0 s.

**TABLE 2:** Patient‐specific dose metrics and delivery time for the nominal robust and PTV plans

				Pt 1	Pt 2	Pt 3	Pt 4	Pt 5	Pt 6	Pt 7	Pt 8	Pt 9	Pt 10	Mean ± SD	*p*‐value
CTV	D2%	[Gy]	Robust	62.4	61.9	62.6	61.7	62.4	61.6	61.8	61.8	62.3	61.7	62.0 ± 0.4	<0.001
			PTV	61.1	61.6	61.6	61.0	62.2	61.1	60.9	61.1	61.1	61.2	61.3 ± 0.4	
	D98%	[Gy]	Robust	59.7	59.5	59.8	59.7	59.9	59.7	59.7	59.7	59.7	59.8	59.7 ± 0.1	<0.001
			PTV	59.9	60.1	60.1	60.0	60.1	59.9	60.1	59.9	60.1	59.8	60.0 ± 0.1	
	HI		Robust	0.044	0.040	0.047	0.033	0.041	0.030	0.034	0.034	0.042	0.032	0.038 ± 0.006	<0.001
			PTV	0.020	0.025	0.025	0.016	0.034	0.020	0.013	0.020	0.016	0.022	0.021 ± 0.006	
	TC	[%]	Robust	96.1	93.2	96.1	92.7	96.6	94.9	94.2	94.6	94.8	94.6	94.8 ± 1.3	0.003
			PTV	96.9	98.7	98.9	98.4	98.5	96.0	99.6	96.5	98.9	94.2	97.7 ± 1.7	
Rectum	V30Gy	[%]	Robust	41.3	37.8	21.7	42.2	35.4	37.8	18.9	33.9	40.3	22.7	33.2 ± 8.8	0.157
			PTV	43.0	39.9	21.8	42.4	35.4	38.9	18.6	33.0	41.2	22.3	33.7 ± 9.3	
	V57Gy	[%]	Robust	9.3	8.5	7.4	14.3	13.3	9.4	3.5	6.8	16.1	4.9	9.4 ± 4.1	0.001
			PTV	11.4	11.0	7.5	16.1	13.6	11.9	4.8	7.6	19.1	5.5	10.9 ± 4.6	
Bladder	V40Gy	[%]	Robust	37.8	26.7	18.9	11.4	23.5	12.8	18.7	17.7	36.8	22.1	22.6 ± 9.0	0.010
			PTV	37.9	28.9	19.3	12.1	25.7	12.9	19.3	18.3	38.8	22.2	23.5 ± 9.3	
	V50Gy	[%]	Robust	26.6	16.7	13.2	8.5	15.8	7.9	12.2	11.5	24.3	15.1	15.2 ± 6.1	<0.001
			PTV	27.9	19.0	13.4	9.0	17.6	8.7	13.7	12.6	26.5	16.7	16.5 ± 6.6	
	V60Gy	[%]	Robust	9.5	4.4	4.7	3.2	5.0	1.7	4.1	3.2	6.0	5.6	4.7 ± 2.1	<0.001
			PTV	10.3	5.9	5.1	3.2	6.3	2.4	5.3	3.7	8.1	6.8	5.7 ± 2.4	
Delivery time		[sec]	Robust	280	264	251	231	250	225	249	253	297	224	252.4 ± 23.4	<0.001
			PTV	290	285	267	248	269	236	252	272	313	236	266.8 ± 24.7	

Table 3 shows the data for each metric of the worst perturbed doses in the evaluations of robustness; the patient‐specific metrics are shown in Tables S1–S4.

**TABLE 3:** Dose metrics (means and standard deviations) for worst‐case perturbed plans with varying setup errors and anatomical change

			5/3 mm setup error			8/5 mm setup error			10/7 mm setup error			Organ motion		
			Robust	PTV		Robust	PTV		Robust	PTV		Robust	PTV	
			Mean ± SD		*p*‐value	Mean ± SD		*p*‐value	Mean ± SD		*p*‐value	Mean ± SD		*p*‐value
CTV	D2%	[Gy]	62.2 ± 0.3	61.6 ± 0.5	0.003	62.4 ± 0.3	61.7 ± 0.5	0.002	62.4 ± 0.3	61.8 ± 0.5	0.003	62.5 ± 0.2	61.6 ± 0.5	<0.001
	D98%	[Gy]	59.6 ± 0.1	59.8 ± 0.1	0.007	59.5 ± 0.1	59.6 ± 0.2	0.410	58.1 ± 0.7	57.6 ± 1.2	0.277	59.5 ± 0.2	59.7 ± 0.2	0.062
	HI		0.041 ± 0.005	0.026 ± 0.008	<0.001	0.046 ± 0.004	0.032 ± 0.009	0.002	0.070 ± 0.009	0.069 ± 0.020	0.800	0.046 ± 0.003	0.030 ± 0.010	0.001
	TC	[%]	93.2 ± 1.9	92.5 ± 3.4	0.510	92.2 ± 2.2	84.4 ± 7.5	0.009	88.6 ± 2.4	74.8 ± 10.6	0.003	92.6 ± 2.0	95.6 ± 2.1	0.005
Rectum	V30Gy	[%]	43.5 ± 10.9	44.3 ± 11.5	0.007	48.8 ± 12.4	49.8 ± 12.7	<0.001	52.0 ± 13.2	53.1 ± 13.2	<0.001	37.8 ± 10.1	38.3 ± 10.1	0.109
	V57Gy	[%]	20.3 ± 7.0	22.5 ± 7.7	<0.001	27.6 ± 8.6	29.9 ± 9.3	<0.001	32.2 ± 9.5	34.7 ± 10.3	<0.001	16.4 ± 7.4	17.8 ± 7.8	<0.001
Bladder	V40Gy	[%]	31.1 ± 10.3	32.7 ± 10.8	<0.001	36.0 ± 10.9	37.7 ± 11.1	0.001	39.4 ± 11.2	41.3 ± 11.7	<0.001	26.5 ± 10.3	27.4 ± 10.7	0.009
	V50Gy	[%]	22.2 ± 7.7	23.8 ± 8.1	<0.001	26.4 ± 8.2	28.4 ± 8.7	<0.001	29.3 ± 8.6	31.5 ± 9.0	<0.001	19.3 ± 7.7	20.7 ± 8.1	<0.001
	V60Gy	[%]	9.2 ± 3.6	10.5 ± 3.7	0.002	12.0 ± 4.3	13.3 ± 4.6	0.003	14.0 ± 4.8	15.4 ± 5.3	0.004	8.5 ± 3.9	9.6 ± 4.1	0.001

The HI values of the PTV plans were better than those of the robust plans in both setup‐error scenarios, but no significant difference was observed in the 10/7 mm setup error scenario. In contrast to the nominal scenario, the TC values of the robust plans were higher than those of the PTV plans in the worst scenarios, considering the setup errors more than the PTV margin. The values increased by 6.9% and 12.2% for the 8/5 mm and 10/7 mm‐setup errors, respectively. Meanwhile, there was no significant difference in the TC value for 5/3 mm setup error although the mean value in robust plans was higher. The OAR doses of the robust plans were lower under both setup errors, and significant differences were observed for all OAR dose metrics. However, a benefit from robust plans for TC was not observed in the worst scenario, considering anatomical changes.

**FIGURE 2:**
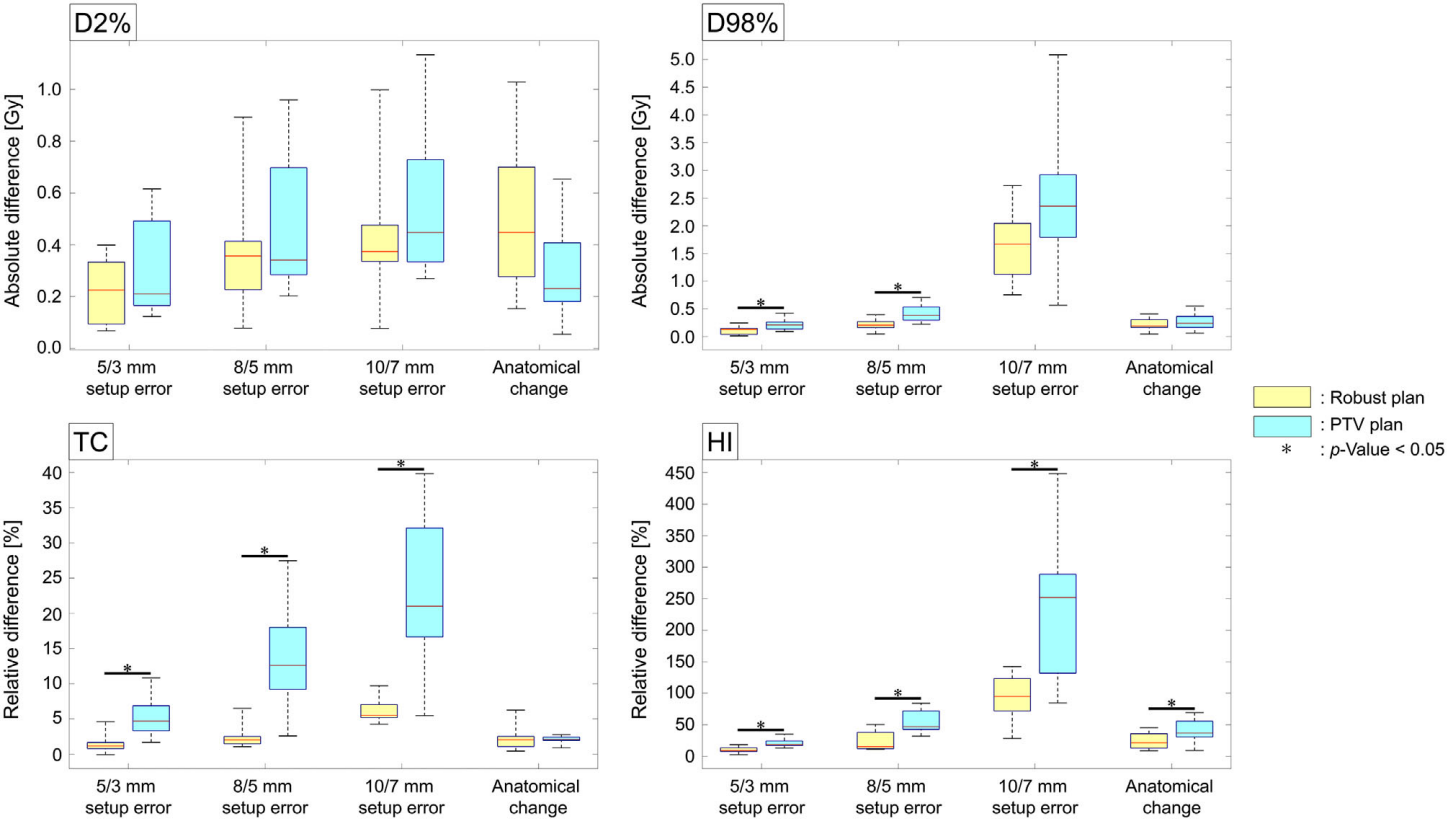
Boxplots of the differences in CTV dose metrics between nominal and worst‐case perturbed plans, including D2%, D98%, homogeneity index (HI) and V100% (TC). The robust plans (yellow) and PTV plans (sky‐blue) are shown. The asterisk symbols indicate *p*‐values < 0.05.

Figure 2 shows boxplots for the difference of CTV D2%, D98%, HI, and TC between the nominal plan and each worst‐perturbed plan for 10 patients. The deterioration rates of HI and TC against the three types of setup errors were significantly lower in the robust plans compared to those in the PTV plans (p < 0.05). Against anatomical change, there was a significant difference between the robust and PTV plans in only the HI deterioration rate.

**FIGURE 3:**
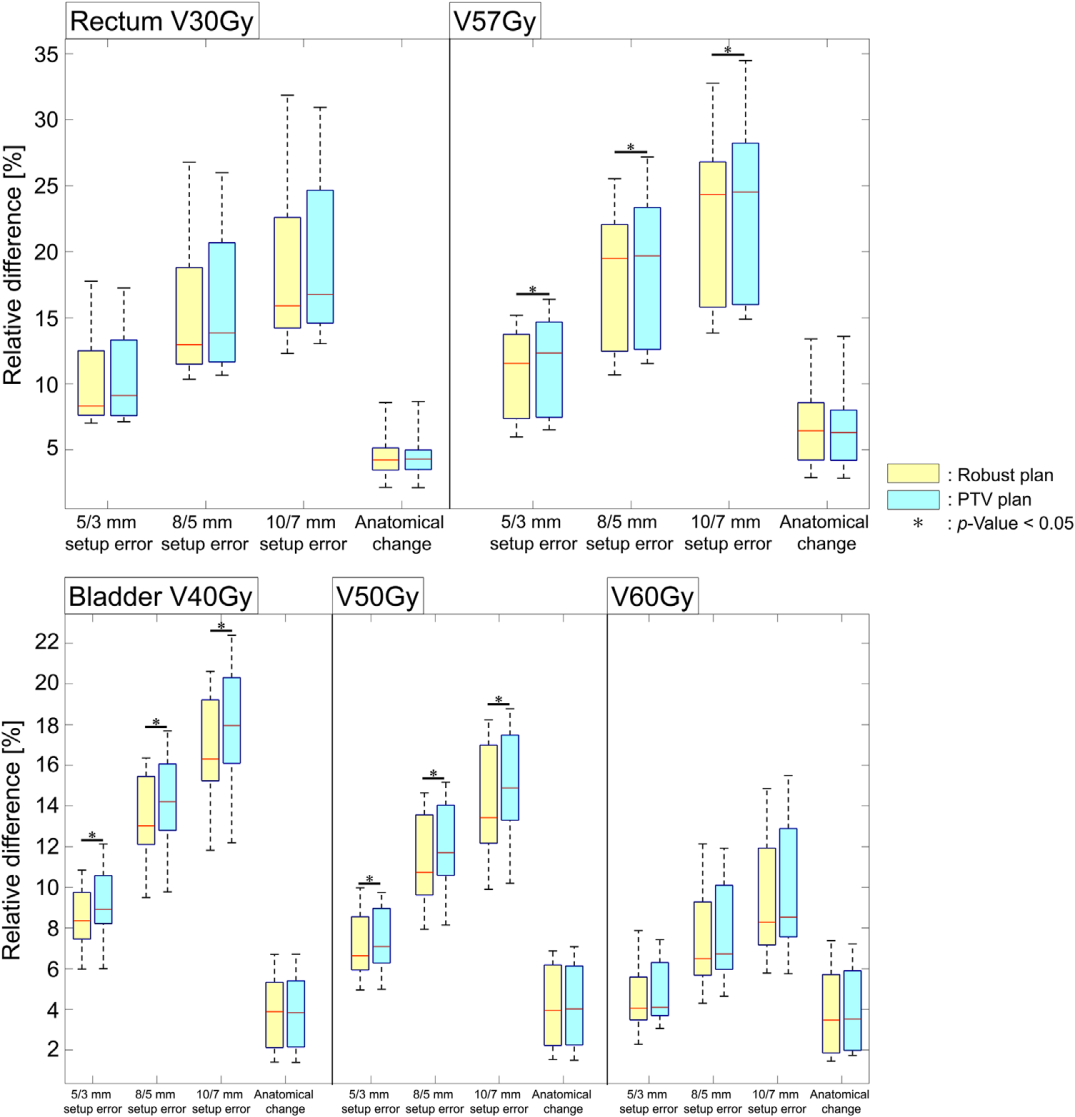
Boxplots of the differences in OAR dose metrics (rectum V30Gy and V57Gy; bladder V40Gy, V50Gy and V60Gy) between nominal and worst‐case perturbed plans. The robust plans (yellow) and PTV plans (sky‐blue) are shown. The asterisk symbols indicate *p*‐values < 0.05

Boxplots of the relative difference in OAR dose metrics for the 10 patients are shown in Figure 3. There were significant differences between the robust and PTV plans in the deterioration rates against setup errors of OAR doses for the bladder V40Gy and V50Gy and the rectum V57Gy (p < 0.05).

Tables S5–S8 show the patient‐specific deterioration rates of HI, TC, and OAR doses between the nominal and worst‐perturbed plans.

## 4 DISCUSSION

The dosimetric comparison between the robustly optimized plans and the conventional PTV margin–based plans showed that compared to PTV plans, robust plans can significantly reduce the dose in the intermediate to high range (40–60 Gy) for the bladder and in the high range (57 Gy) for the rectum in nominal and all uncertainty cases. Regarding bladder dosimetry, not only the high dose to the bladder, but also intermediate dose volumes can contribute to urinary toxicities.^34^ In addition, rectal bleeding is the most undesirable toxicity following radiotherapy for prostate cancer. High dose to the rectum is considered strongly associated with rectal bleeding.^35^ Hence, using robust optimization might be an effective approach to decrease the risk of these radiation‐induced toxicities. Selection of patients using normal tissue complication probability model is a promising method to decide which plan would be preferred for the patient.^36^ All nominal robust and PTV plans fulfilled the target coverage requirements, where the near‐minimum doses (D98%) to the CTV were close to the prescription dose. However, the robust plans showed lower target dose homogeneity than the PTV plans. This is because robust optimization tends to create a high‐dose region inside the target. This result is consistent with that of other studies.^8^ ^,^ ^9^ ^,^ ^27^

To evaluate the effect of the robust optimization method on plan robustness, perturbed dose plans accounting for patient‐setup errors were created using both nominal plans. From the result of the 5/3 mm setup error, we found that there was no advantage of robust optimization in terms of TC; nevertheless, the efficacy of OAR sparing of robust optimization remained higher than that obtained using the conventional method. When considering the 8/5 mm setup error, which had the same magnitude as the PTV margin, the mean TC (V100%) was less than 90% in the worst‐case PTV plans. In contrast, the worst‐case robust plans remained above 90% for TC. Moreover, the mean deterioration rate of TC in the worst‐case nominal PTV plans was 21.0% considering a 10/7 mm setup error, whereas that in the worst‐case robust plans was 6.2%. The robust optimization method demonstrated greater robustness even for setup errors larger than the compensation margin. Interestingly, no significant difference in dose homogeneity was observed between the worst‐case robust and PTV plans considering the 10/7 mm setup error. This was because the near minimum doses in the target were considerably decreased due to the lack of compensation performance of the PTV margin under a larger setup error.

Several factors are known to affect the dose distribution in radiotherapy for prostate cancer. The position and shape of the prostate can be changed by variations in intestine, bladder, and rectum filling.^37^ When we evaluated the robustness of the plans against anatomical changes caused by the target variation, the evaluation showed that the deterioration rates of TC, and OAR doses between the nominal and worst‐case plans were comparable to those of the PTV margin method. In helical IMRT plans, traditional 3D robust optimization might be insufficient to account for anatomical changes in the target. Recent studies have shown that including information about anatomical changes such as non‐rigid positioning uncertainties in robust optimization can improve plan robustness.^38^ ^–^ ^40^ Some OAR doses in the worst‐case plans were beyond the clinically acceptable criteria, even for robust plans. The OAR doses caused by uncertainties may be further decreased by applying robust optimization for both the target and OAR objectives in the optimization process.

One finding was that the delivery times of the robust plans were significantly shorter than those of the PTV plans, which may have led to a reduction in patient burden. However, the treatment planning took much longer (up to 1 h per plan) because a calculation of all possible scenario doses was required in each iteration. In contrast, the planning times for the PTV plans were less than 10 min. This is an unavoidable limitation when using robust optimization. Recent studies have reported that using a limited set of relevant uncertainty scenarios in the robust optimization process can reduce the planning time without compromising plan robustness and OAR sparing in proton therapy.^40^, ^41^ This method might be able to accelerate the calculation process in tomotherapy planning. Another limitation is that plan robustness for setup error and anatomical changes was independently evaluated. In future, further dosimetric investigation would be necessary to evaluate which treatment plan would be robust against a combined effect of setup error and anatomical changes. In addition, it is worth noting that the clinical benefits of robust optimization should be confirmed in future cohort or retrospective studies.

## 5 CONCLUSION

Minimax robust optimized helical IMRT plans provided OAR sparing for localized prostate cancer patients compared with PTV margin–based plans. Plan robustness in terms of target coverage was also demonstrated to be more robust in the presence of systematic patient‐setup uncertainties but not against anatomical changes caused by organ variation.

## AUTHOR CONTRIBUTION

Takayuki Yagihashi and Tatsuya Inoue made substantial contributions to the conception of the study. Takayuki Yagihashi, Akihiro Yamano, Shunsuke Suzuki, Wataru Yamakabe, and Naoki Sato made significant contributions to the data analysis and interpretation. Kazumasa Inoue, Hironori Nagata, Motoko Omura, and Tatsuya Inoue made significant contributions to the design of the work and the interpretation of data. Takayuki Yagihashi and Tatsuya Inoue drafted the original manuscript. All authors critically reviewed and revised the manuscript draft and approved the final version for submission.

## CONFLICT OF INTEREST

The authors declare no conflicts of interest.

## Supporting information

Table S1. Patient specific dose metrics of the worst‐case perturbed plans under 5/3 mm setup error

Table S2. Patient specific dose metrics of the worst‐case perturbed plans under 8/5 mm setup error

Table S3. Patient specific dose metrics of the worst‐case perturbed plans under 10/7 mm setup error

Table S4. Patient specific dose metrics of the worst‐case perturbed plans under anatomical change

Table S5. Deterioration values and rates of dose metrics between nominal and the worst‐case perturbed plans under 5/3 mm setup error

Table S6. Deterioration values and rates of dose metrics between nominal and the worst‐case perturbed plans under 8/5 mm setup error

Table S7. Deterioration values and rates of dose metrics between nominal and the worst‐case perturbed plans under 10/7 mm setup error

Table S8. Deterioration values and rates of dose metrics between nominal and the worst‐case perturbed plans under anatomical change

## Data Availability

The data that support the findings of this study are available from the corresponding author upon reasonable request.
