# Supplementary material for: Effectiveness of robust optimization against geometric uncertainties in TomoHelical planning for prostate cancer
Source: J Appl Clin Med Phys. 2022 Dec 28;24(4):e13881. doi: 10.1002/acm2.13881 (PMC10113685; doi:10.1002/acm2.13881)
Supplement: Supplementary file 3 — Table S3. Patient specific dose metrics of the worst‐case perturbed plans under 10/7 mm setup error [file ACM2-24-e13881-s004.docx]

Table S2. Patient specific dose metrics of the worst-case perturbed plans under 8/5-mm setup error
